# Fully-Automatic 3D Intuitive Visualization of Age-Related Macular Degeneration Fluid Accumulations in OCT Cubes

**DOI:** 10.1007/s10278-022-00643-6

**Published:** 2022-05-05

**Authors:** Emilio López-Varela, Plácido L. Vidal, Nuria Olivier Pascual, Jorge Novo, Marcos Ortega

**Affiliations:** 1 Grupo VARPA, Instituto de investigación Biomédica de A Coruña (INIBIC), Xubias de Arriba, 84, A Coruña, 15006 Spain; 2 Centro de investigación CITIC, Universidade da Coruña, Campus de Elviña, s/n, A Coruña, 15071 Spain; 3 Servizo de Oftalmoloxía, Complexo Hospitalario Universitario de Ferrol, CHUF, Av. da Residencia, S/N, Ferrol, 15405 Spain

**Keywords:** Computer-aided diagnosis, 3D visualization, Optical Coherence Tomography, Age-related macular degeneration

## Abstract

Age-related macular degeneration is the leading cause of vision loss in developed countries, and wet-type AMD requires urgent treatment and rapid diagnosis because it causes rapid irreversible vision loss. Currently, AMD diagnosis is mainly carried out using images obtained by optical coherence tomography. This diagnostic process is performed by human clinicians, so human error may occur in some cases. Therefore, fully automatic methodologies are highly desirable adding a layer of robustness to the diagnosis. In this work, a novel computer-aided diagnosis and visualization methodology is proposed for the rapid identification and visualization of wet AMD. We adapted a convolutional neural network for segmentation of a similar domain of medical images to the problem of wet AMD segmentation, taking advantage of transfer learning, which allows us to work with and exploit a reduced number of samples. We generate a 3D intuitive visualization where the existence, position and severity of the fluid were represented in a clear and intuitive way to facilitate the analysis of the clinicians. The 3D visualization is robust and accurate, obtaining satisfactory 0.949 and 0.960 Dice coefficients in the different evaluated OCT cube configurations, allowing to quickly assess the presence and extension of the fluid associated to wet AMD.

## Introduction

Age-related macular degeneration (AMD) is a progressive and degenerative disease of the central retina, which can lead to a significant loss of the central vision. It affects approximately 8.7% of the population of the world, being the leading cause of irreversible vision loss in industrialized countries and the third leading cause of blindness worldwide [1–3]. While there are many subtypes of AMD, they can essentially be grouped into two: non-neovascular, known as dry AMD, and neovascular, known as wet AMD [4]. Dry-type AMD is the most common and mildest form of the disease, characterized by a gradual destruction of the retinal pigment epithelium and the photoreceptors. About 20% of the cases of this type of AMD are transformed into wet-type AMD [5]. Although wet AMD is less common than its dry variant, it is present in 80% of the patients suffering from AMD with severe vision loss [6]. In addition, several studies have linked wet AMD to a significant decrease in the quality of life and have identified wet AMD as a risk factor for depression [7]. Wet-type AMD is the most severe and aggressive form of the disease, in which fluid appears near the retinal layer due to the creation of new thin-walled capillaries that filter fluid into the macula. The resulting scarred retina significantly and irreversibly reduces the visual capacity, therefore an early detection is critical [8, 9]. Although several treatments exist, the current main treatment for wet-type AMD are invasive intravitreal injections of anti-vascular endothelial growth factor (anti-VEGF) agents [10].

**Fig. 1:**
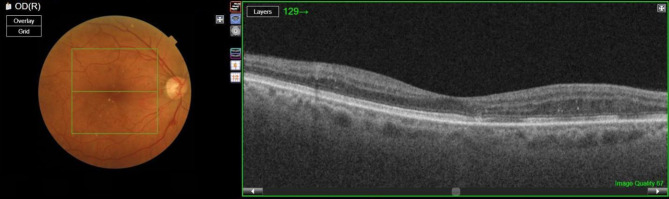
On the left, the fundus image with the analysis area marked and a green line marking the particular slice. On the right, the OCT slice is shown

Currently, the main technique used in the diagnosis of this disease is optical coherence tomography (OCT), an in vivo non-invasive technique based on the different reflectance of the layers that are present in the ocular structure that allows the acquisition of transversal images of the retina with semihistological resolution [11]. As a result of an OCT test, a sequence of 2D slices is generated which together form a 3D volume. An example of the interface of an OCT device is shown in Fig. 1. It shows an image of the fundus and a particular cross section.

**Fig. 2:**
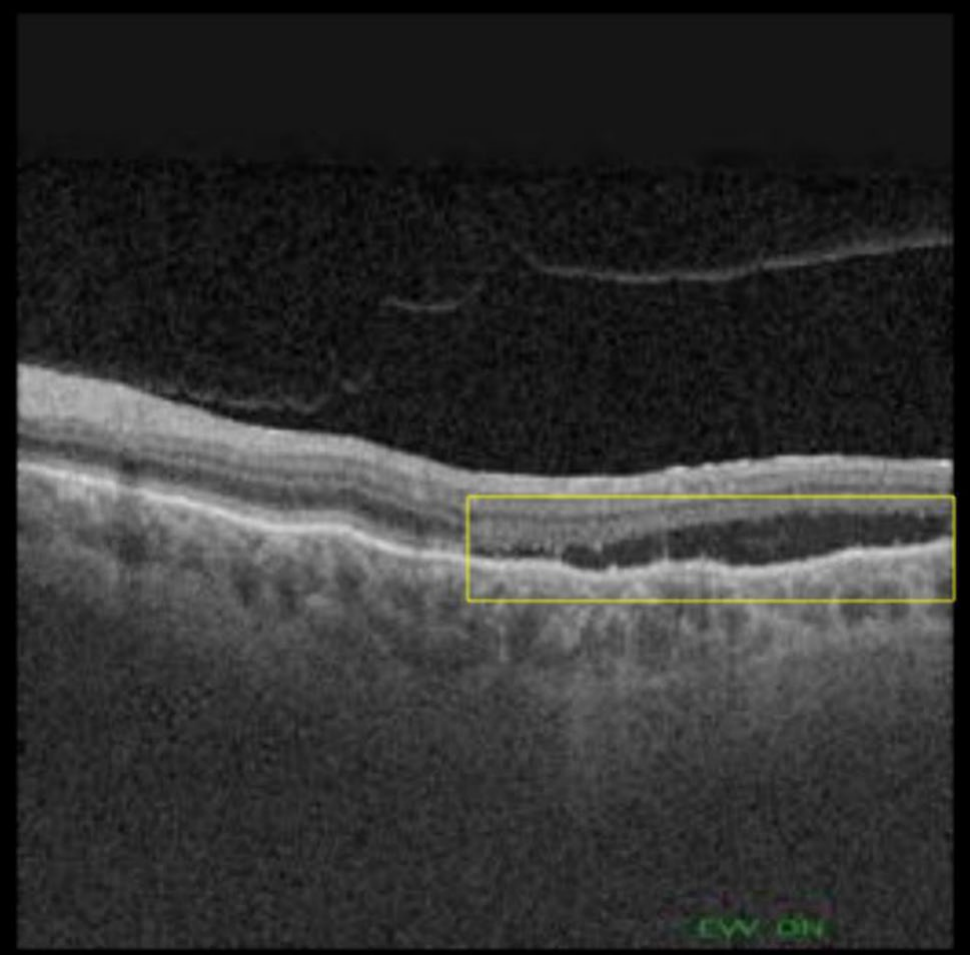
OCT image showing subretinal fluid associated with AMD (surrounded by a yellow box)

Each OCT exam results in hundreds of scans that must be analyzed by a physician, involving a process influenced by the subjectivity and experience of the physician [12]. The likelihood of human error associated with these factors can be alleviated with the development of a fast and reliable automated system that can detect and visualize the presence and extent of the fluid associated with wet AMD. A representative example of the fluid accumulations present in a patient with wet AMD is shown in Fig. 2.

The problem of the automatic AMD-associated fluid segmentation in OCT images has been explored using different methods and from different perspectives of use. One of the first works on wet AMD fluid segmentation in OCT images was done by Fernández [13] and consisted of a semi-automatic 2D approach based on active contours. A similar approach was later used by Zheng et al. [14]. One of the first works with an automated approach was proposed by Quellec et al. [15], where information based on retinal texture and thickness properties were used.

From the perspective of deep learning, there are several works focused on the segmentation of the different appearances of fluid present in the OCT images (they may be present by AMD or other diseases) with variations in their methodologies but mainly for 2D analysis of particular histological cuts of the entire 3D OCT scans. In the state of the art, the works that use the U-net [16] architecture or some of its variants stand out. Thus, in the work of Lee et al. [17], a variant of the U-net was used to perform a binary segmentation of the intraretinal fluid. In the work of Venhuizen et al. [18], an approximation to the problem was made using two U-nets simultaneously: the first delimited the retinal region, while the second used this information to segment the cystoid fluid associated with AMD. In the work of Rashno et al. [19], a convolutional network and Graph Shortest Path were used to segment fluids in subjects with AMD and diabetic macular edema. In the work of Tennakoon et al. [20], an adversarial network was used during the training of a U-net adaptation to code higher order relationships between image regions and segment retinal fluids in subjects with AMD. Finally, Lu et al. [21] applied another U-net variation and a random forest classifier for multiclass retinal fluid segmentation.

As seen, the vast majority of strategies are based on a 2D analysis of particular histological cuts, which can reflect only a certain aspect of the body tissue information. The physician needs to infer the 3D structure of the pathological tissue of the entire 3D OCT through the two-dimensional representation according to his or her clinical knowledge and experience, which limits the use of medical imaging in the clinical diagnostic process and is prone to be affected by the subjectivity of the clinician. Three-dimensional reconstruction allows physicians to make a more accurate and intuitive diagnosis, which reduces their subjectivity in the diagnostic process and therefore greatly improves the accuracy of the medical diagnosis [22].

Regarding the 3D visualization of OCT images, most of the works deal with the visualization of blood capillaries such as Athanasiou et al. [23], Zhang et al. [24] and Spaide [25]. Works dealing with 3D AMD fluid visualization are much scarcer. In the work of Bower et al. [26], a manual segmentation of the photoreceptor layer and drusen was performed and a 3D reconstruction of these was done independently in order to easily visualize the factors associated with dry AMD. In the work of Chen et al. [27], an automated graph-theory method was used to create a 3 colours display where the fluid between the upper and lower layers of the retina was shown.

Although there are works that deal with fluid segmentation in OCT images, most of them work using particular 2D cuts, while our work focuses on the entire 3D volume of the OCT scan. Besides, all these works are usually focused on one of these two objectives. On one hand, to improve the segmentation algorithm itself, either for fluid associated to AMD, fluid in general or to catalogue different subtypes of fluid. On the other hand, to use the segmentation to obtain a biomarker. In contrast, the objective of our work is to use the segmentation with the ultimate goal of offering the physician a clear and intuitive 3D visualization that will allow the physician to diagnose at a glance if a patient has AMD and its severity. As previously stated, the diagnosis and monitoring of the evolution of wet AMD is a time-consuming, exhausting and prone to subjectivity process. Therefore, an automatic system is highly desirable to help the physician to make an early diagnostic, critical to prevent irreversible vision impairment in this disease. To the best of our knowledge, there is no system with these specific characteristics. In summary, the main contributions of this article are:

- Proposal of a fully automatic methodology to segment the intraretinal and subretinal fluid associated with AMD in OCT images.
- Robust and representative methodology, trained and tested with different characteristic configurations of 3D OCT volumes.
- Use of deep learning and transfer learning to take advantage of information from a similar medical imaging domain, allowing for the use of a reduced number of images to achieve an adequate performance.
- Intuitive and coherent 3D visualization of the fluid associated with AMD that facilitates the work of doctors and allows a robust diagnosis, independently of the subjectivity of the physician.
- Interactive 3D visualization that allows the physician to view the entire 3D volume from any needed angle, instead of viewing the 2D slices individually.

## Material and Methods

### Dataset

The dataset consists of a total of 4,832 images obtained from 48 OCT volumes. 42 of these OCT volumes are from patients who present AMD and 6 from control patients who do not present AMD. All the OCT cubes were taken using an OCT capture device DRI OCT Triton from Topcon Corp.

**Fig. 3:**
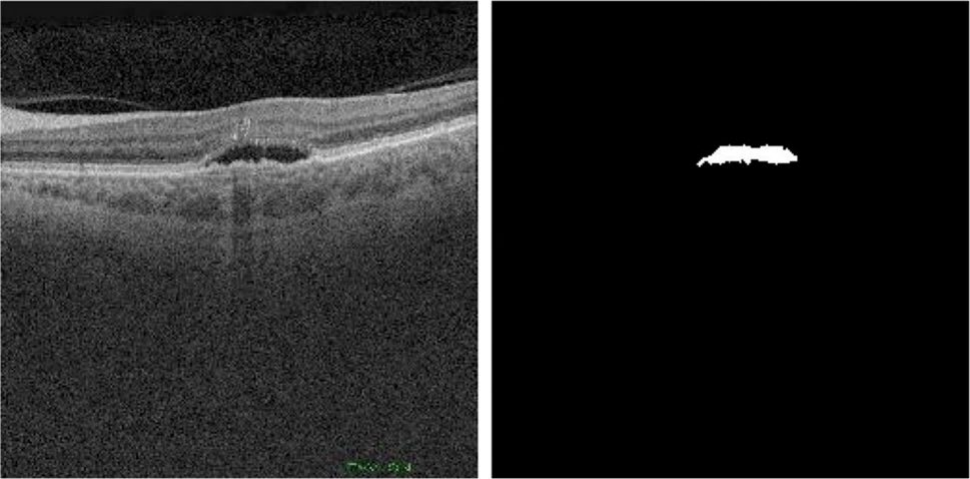
Example of OCT image (left) and corresponding mask (right)

Within this set of cubes, there are two different configurations of volumes. The first type of volume, that we will now call OCT256, consists of 256 cross-sectional OCT images with a resolution of 512 × 992. This configuration is the most common in OCT volumes obtained by Triton. The second type, that we will now call OCT320, consists of 320 cross-sectional OCT images with a resolution of 320 × 992 pixels. These volumes are obtained as an intermediate by-product of a frequently taken ophthalmological test. As shown in Fig. 3, each image includes the corresponding ground-truth mask associated with it, where all the pixels were labeled as background (black) or fluid (white) by an expert.

### Software Resources

Regarding the software resources, we used Python 3.7.9 with PyTorch 1.6 framework [28] and the VTK 9.0.1 open source library [29]. Additionally, a pre-trained U-net model from the work of Buda et al. [30] was used, trained with images from 110 patients for a total of 7,858 images.

### Methodology

**Fig. 4:**
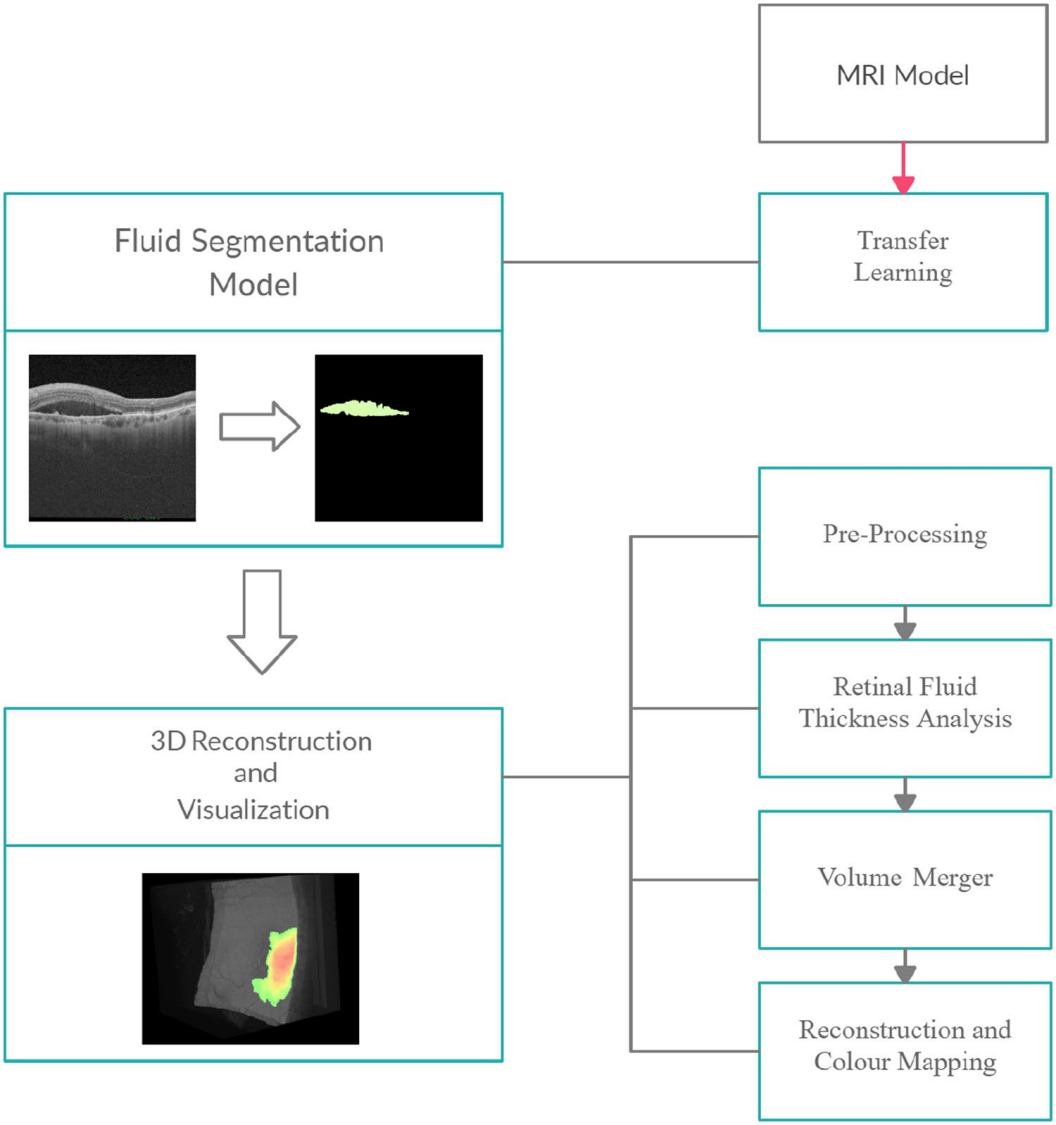
Diagram showing the methodology used to carry out the final 3D reconstruction

In order to develop a system capable of adequately segment the 3D fluid and provide an intuitive 3D reconstruction, our methodology was divided into two main stages. First, we exploit the capabilities of deep learning and transfer learning in order to train with a reduced number of samples a model capable of effectively segment the fluid associated to wet AMD. Then, the creation of an intuitive 3D visualization that allows at a glance to identify the 3D presence and extent of the fluid associated to wet AMD. A diagrammatic summary of the methodology associated with these sections can be seen in Fig. 4.

### Transfer Learning

We first started by using a model previously trained in a similar medical imaging domain such as MRI. This model has been trained with a large number of images for the task of glioma (a type of brain tumor) segmentation. The choice of this particular model was due to the similarities present between our type of image and the images belonging to the magnetic resonance modality as well as the similarity between the patterns of the pathologies, which implies that this model is closer to the convergence than a model initiated randomly and allows us to train and achieve an adequate performance with fewer images. In addition, the U-net model has proven its robustness and reliability in tasks related to our target medical imaging field in numerous state-of-the-art works. In this way, we use transfer learning to continue the training of the network using our OCT image dataset to solve our specific segmentation task.

### Training of the Models

All the images in the dataset were resized to $256 \times 256$ pixels to fit the pre-trained network. The dataset was divided into the training and validation dataset and the test dataset. The training and validation dataset consists of a total of 1,056 (942 images in the training set and 114 images in the validation set) images obtained from 35 OCT volumes of eyes with AMD. 20 of these volumes were used to obtain the images of the training set and the other 15 were used to obtain the images of the validation set. The remaining 13 OCT volumes, independent of the training and validation process, were used as whole cubes (a total of 3,776 images) in the test dataset. Within these 13 cubes, 6 volumes belong to the OCT256 group while the other 7 volumes belong to the OCT320 group. Additionally, in the 6 volumes from OCT256 and in the 7 volumes from OCT320, we have guaranteed the presence of at least and at most of two control patients (cubes from healthy individuals) used to evaluate if our system is robust against false positives.

In this work, the Smooth Dice loss was used as a loss function when training the network (1). Smooth Dice loss works much better than other standard metrics such as cross entropy [31] when using imbalanced datasets. Thus, in our images, there was a general imbalance between fluid and background classes, the latter being much more common.

1 $$\begin{aligned} SmoothDiceLoss(X,Y)= 1 - 2\frac{(X \cap Y) + 1}{X + Y + 1} \end{aligned}$$

A batch size of 10 was used as it offered the best results in previous tests. As an optimizer, we used the stochastic gradient descent (SGD) with an initial learning rate of 0.001 using the momentum strategy of Nesterov [32]. It used a dynamic learning rate that was reduced by a factor of 0.3 if the loss of validation did not fall after 40 epochs. Additionally, an early stopping function was used which stopped the training if the validation loss did not drop for 80 epochs in a row, always keeping the model with the best validation loss.

**Fig. 5:**
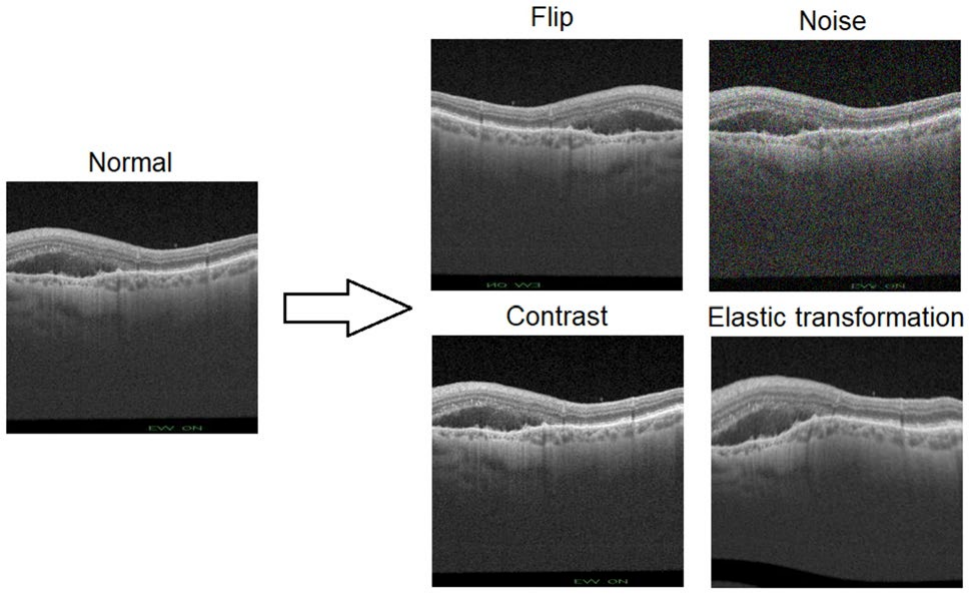
Individual examples of each transformation on the same image

**Fig. 6:**
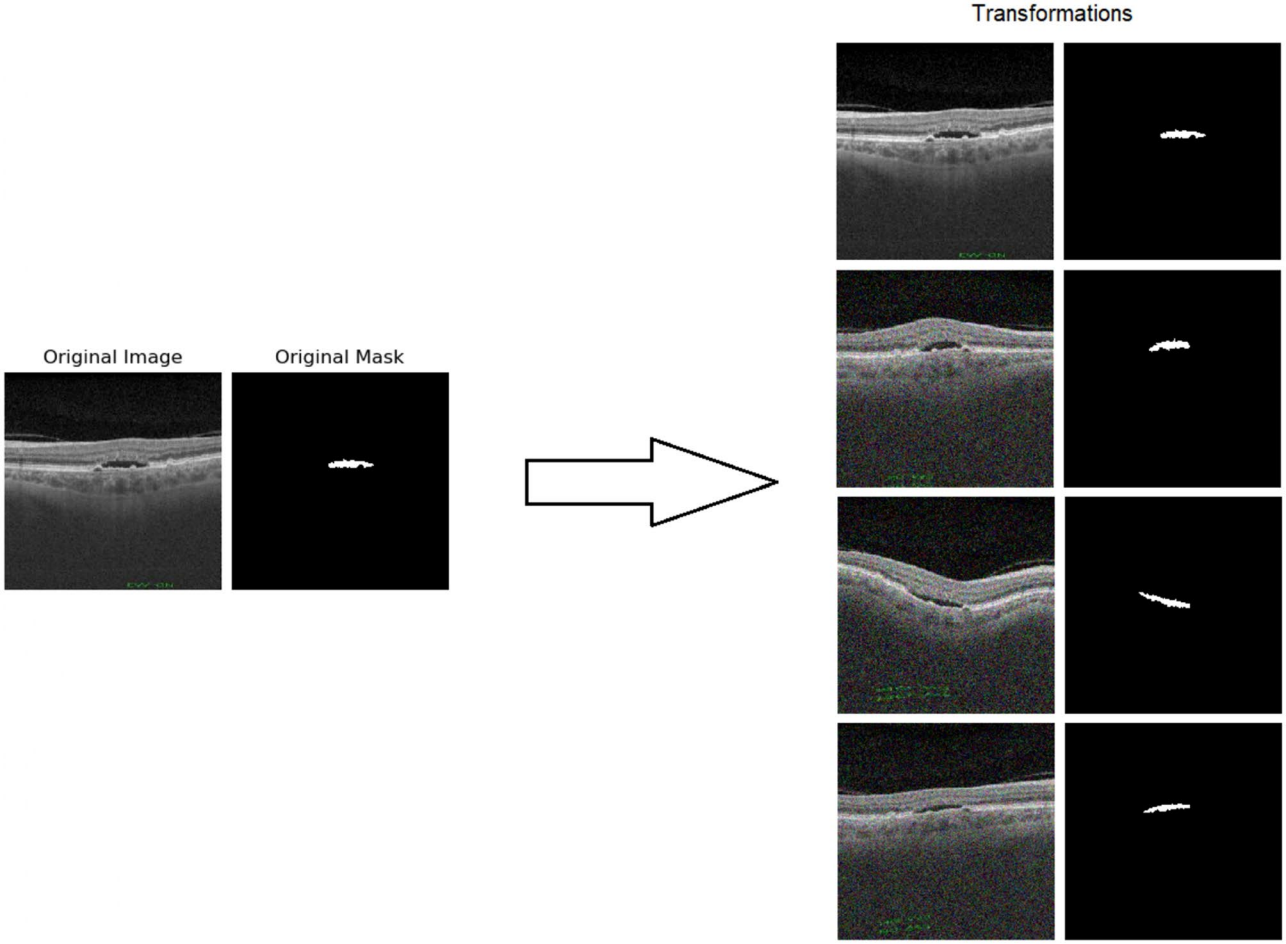
Several examples of the overall application of all the transformations on the same image. The first 2 images correspond to the original image and the original mask

To make the model more robust and avoid overfitting and optimize the performance with the reduce presented of samples to the network, data augmentation techniques have been also implemented. The images undergo a series of online transformations with a 50% of probability. In each batch, the sampled images of the training set undergo a horizontal flip. In addition, noise was added to the image. The different considered types of noise were Gaussian, salt and pepper or speckle type. After that, an elastic transformation [16] was applied. The elastic transformation is particularly interesting in this problem, since the elastic transformations imitate the possible deformations that both normal and pathological retinas usually present. Finally, the contrast of the image was randomly changed. A graphic example of each of these transformations can be seen in Fig. 5. Several examples of the overall application of all these transformations can be seen in Fig. 6.

### Evaluation

Two different metrics were used to evaluate the quality of the segmentation predicted by our network in a quantitative way. Both metrics measure the degree of similarity between two sets. The first metric used to evaluate the quality of our segmentation is the Dice coefficient (DSC) [33] (2). The intersection over the union (IoU) [34], also known as the Jaccard index (3), is also used.

2 $$\begin{aligned} D(X,Y)= \frac{2*(X \cap Y)}{X + Y} \end{aligned}$$

3 $$\begin{aligned} IoU(X,Y)= \frac{X \cap Y}{X \cup Y} \end{aligned}$$

### 3D Reconstruction and Visualization

Several processing steps were applied to the images and masks predicted by our model in order to produce an accurate and intuitive visualization.

- Pre-Processing: The first step of this processing was a smoothing of the binary masks. The function of this step was, on one hand, to smooth the edges of the fluid and, and on the other hand, to improve the segmentation and the resulting 3D model by mitigating possible artifacts resulting from the segmentation. An example of these artifacts can be protrusions (in areas that should be softer) that represent false positives. This smoothing can be done either by applying a closing or an opening. On one hand, the closing could improve the segmentation by slightly increasing the size of areas that should be larger, thus removing some false negatives. On the other hand, an opening could improve segmentation by removing small false positives present in the images, but could also worsen it by removing small regions of fluid that are true positives.
- Retinal Fluid Thickness analysis: The distance transform was applied on each slice of the generated masks cube to obtain an estimation of the fluid thickness present in a particular OCT image.
- Volume Merger: The cube containing the images and the cube containing the masks that were predicted by our network were merged. The original cube kept the values of its pixels where the generated mask cube had a value of 0. The value of these pixels was within the range [0, 255]. On the other hand, the rest of the pixels adjusted their value in the range [256, 510] based on the value the pixel had in the distance-transformed cube. Subsequently, this allowed the cube to maintain its original appearance in the voxels where there was no fluid, while also containing the fluid thickness information to be used in the reconstruction transfer function.
- Reconstruction and Colour Mapping: Once the fused cube was obtained, the ray casting algorithm was used to obtain the final representation. A linear interpolation was used and the transfer function was adjusted to give color to the pixels where there was fluid. To do so, a Red-Green color scale was used, where red values represent a greater fluid presence. The selection of this color scale is common in programs used by clinicians and the color gradient helps the clinicians to see differences between the different levels of severity more clearly. In the composition process, the maximum intensity (maximum value found in the scalar values of a given ray) was used to obtain the intensity value of each pixel.

## Results and Discussion

### Evaluation of the Segmentation

**Fig. 7:**
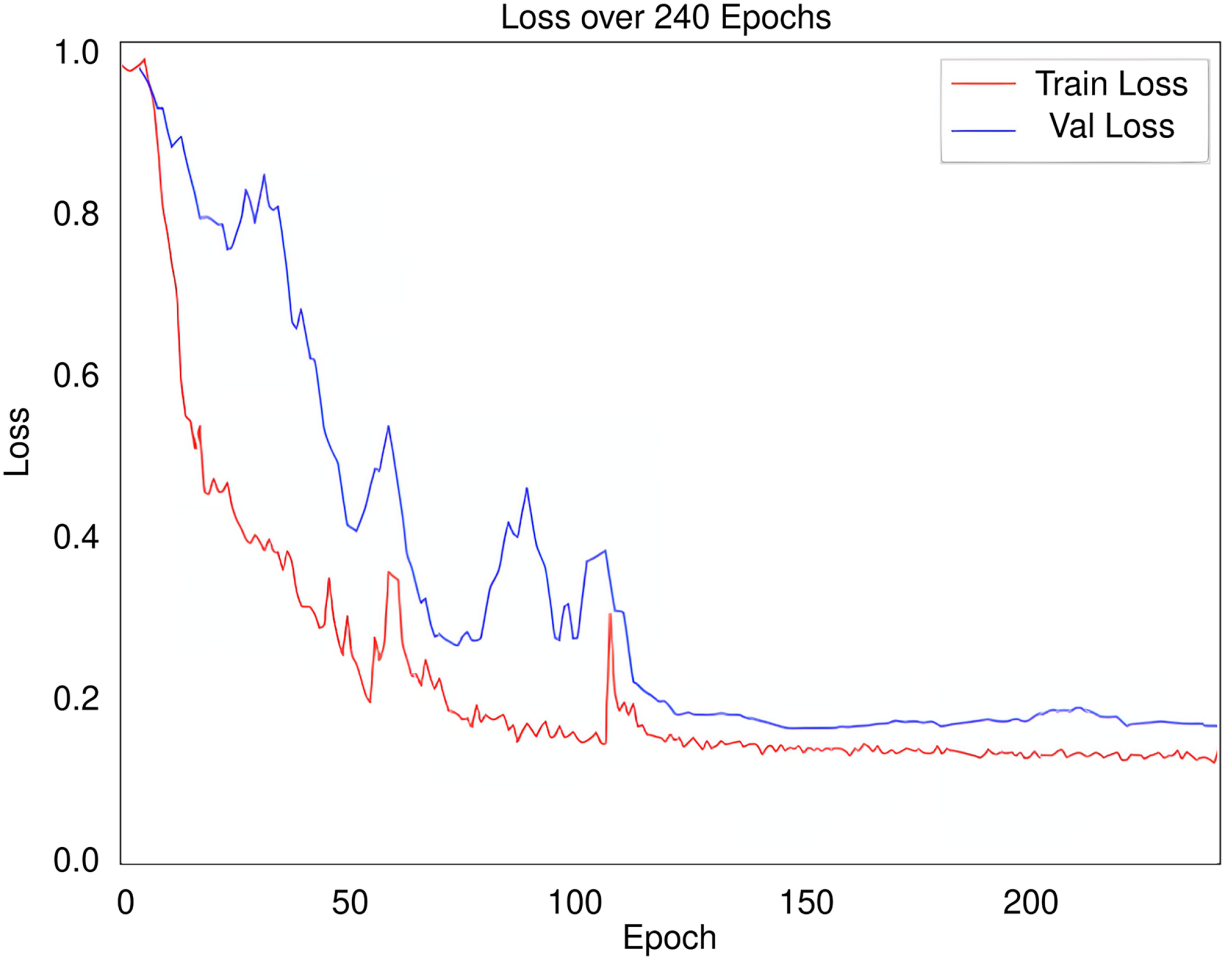
Evolution of the training loss in red and the validation loss in blue throughout the training process

The evolution of the loss in the training and validation sets during the training epochs is shown in Fig. 7. The training of our network ended due to the early stopping and we kept the model with the best validation loss.

In order to see if the quality of the segmentation is affected by the type of used volumes, we calculated DSC and IoU separately for the test cubes belonging to the OCT256 group as well as those belonging to the OCT320 group. Table  1 summarizes the values that were obtained for the metrics calculated in the OCT256 and OCT320 configurations.

**Table 1:** Values obtained in the segmentation evaluation metrics for OCT256 and OCT320

**Metrics**	**OCT256**	**OCT320**
**DSC**	0.9492	0.9603
**IoU**	0.9033	0.9205

**Fig. 8:**
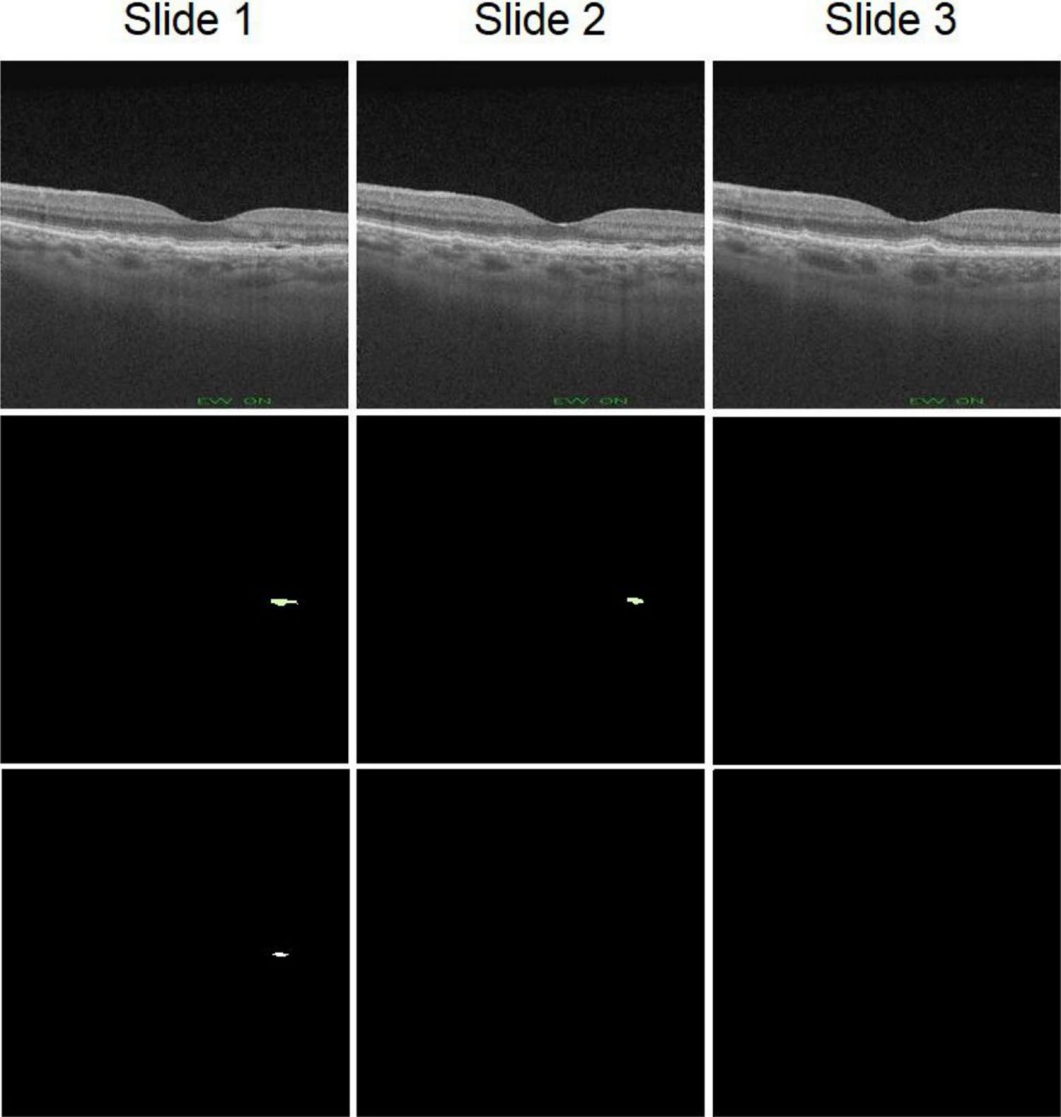
Example showing network segmentation errors in three successive slices with a transition to non-fluid areas. Row one shows 3 close slices of a cube, row 2 shows the masks made by the expert for these slices and row 3 shows the prediction made by the network

The DSC and IoU values indicate a high degree of similarity between the masks created by the expert and those predicted by the model, which implies that the segmentation is faithful to the ground truth. We can see that the cubes of the OCT320 group present a slightly better segmentation performance than those of the OCT256 group. This is due to the fact that the OCT256 group presents a greater number of slices with small regions of fluid than the OCT320 cubes. These regions are segmented precisely by the network, except in the transition slices to a zone without fluid. These transition areas of small regions present a diffuse limit that is difficult to establish, giving rise to small differences between the masks created by the expert and the masks predicted by our network, as it is shown in Fig. 8. In addition, OCT320 has a higher resolution of slices which implies wider and less diffuse transition margins than the lower resolution OCT256 volumes. The cubes of healthy patients present a perfect segmentation, since there are no false positives. Thus showing that the network is robust when it comes to detect the absence of the disease in healthy subjects.

**Fig. 9:**
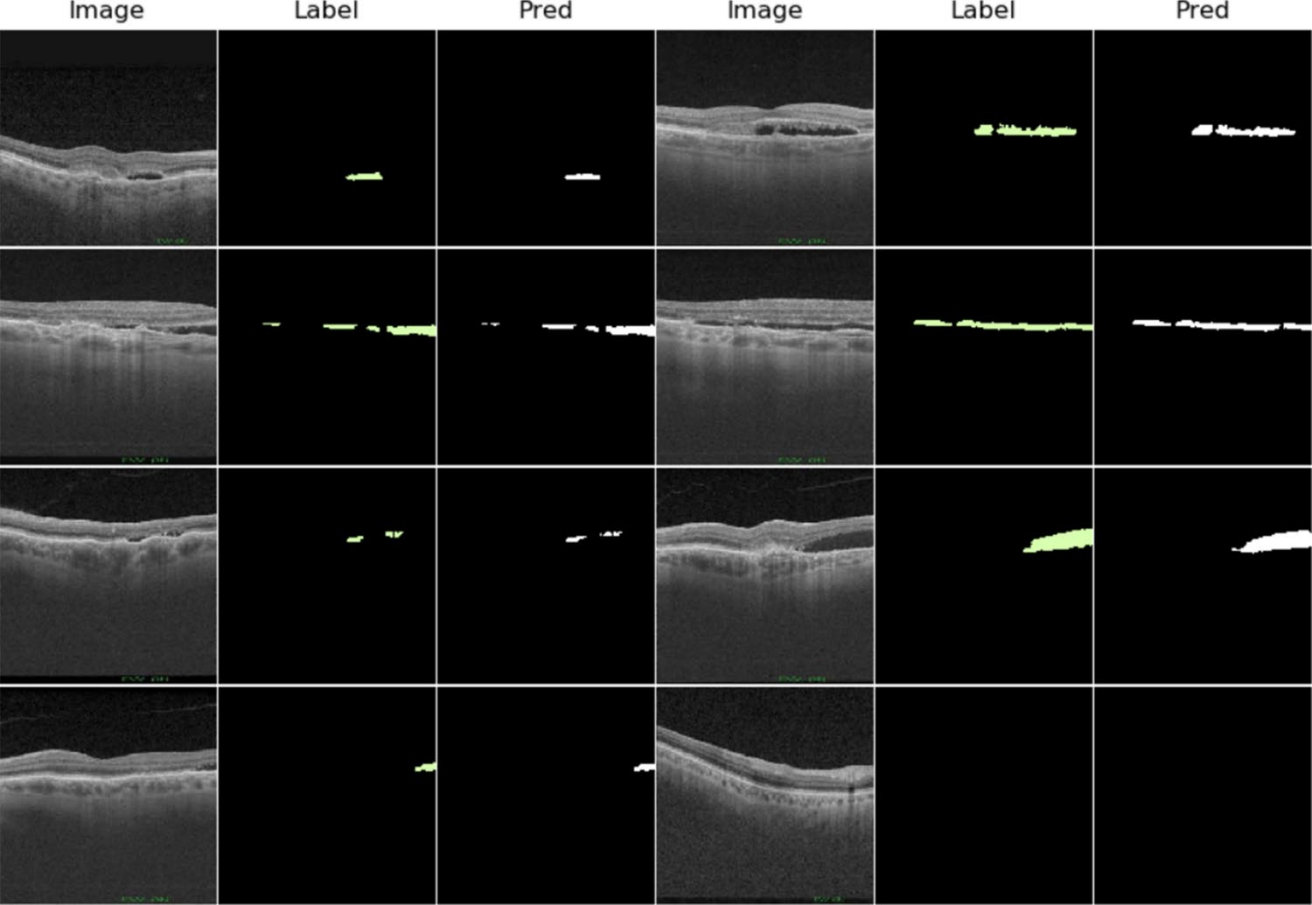
Representation and comparison between some images (Image columns) of the test cubes, the segmentation made by the human expert (Label columns) and the mask predicted by the network (Pred columns)

Complementarily, a qualitative comparison of the segmentation carried out by our proposal with respect to the masks produced by the human expert can be established in Fig. 9.

### 3D Reconstruction and Visualization

**Fig. 10:**
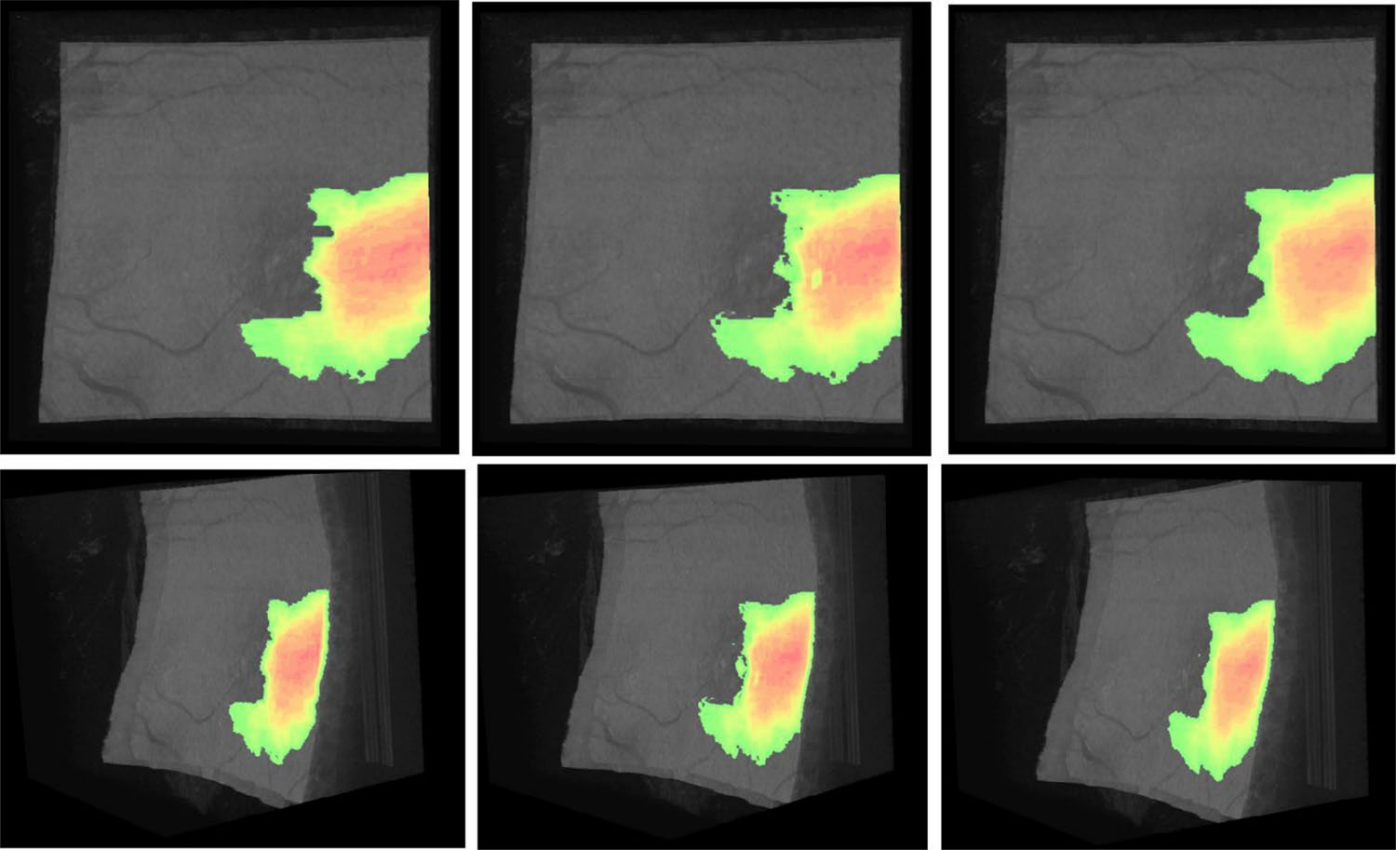
3D reconstruction of the fundus of the patient. The fluid associated with AMD is marked in the color range from red to green. From left to right: cube reconstructed with the masks annotated by the expert, cube reconstructed with the masks predicted by the net and cube reconstructed with the masks predicted by the net using the smoothing

As explained in "3D Reconstruction and Visualization", before carrying out the 3D reconstruction, several processing steps were performed. The first pre-processing step involves the use of a closing or opening operator to smooth out the protrusions from the segmentation. To decide whether to apply a closing or an opening, we calculated independently the similarity metrics without applying smoothing methods. In Table  2, we can see the metrics obtained in each case. Comparing them, we can see that the closing slightly improves the similarity metrics while the opening reduces it. Our network detects a negligible number of false positives as we can see from the healthy cubes and the images of the pathological cubes in which there is no fluid present. With this in mind, we can infer why the opening does not improve the similarity metrics: There are almost no false positives to eliminate and only the negative effects are seen. The application of the opening worsens the segmentation by removing very small fluid present mainly in the transition areas where the network has more problems. On the other hand, the closing operation produces a small improvement in the similarity metrics, improving the segmentation in these small specific areas where the edges are diffuse. For all these reasons we finally chose the closing to be included in our preprocessing strategy. The effect of applying the closing operation on the 3D visualization is shown in Fig. 10.

**Table 2:** Values obtained in the segmentation evaluation metrics for OCT256 and OCT320 and in OCT256 and OCT320 after applying a closing or an opening

**Metrics**	**OCT256**	**OCT256 Close**	**OCT256 Open**	**OCT320**	**OCT320 Close**	**OCT320 Open**
**DSC**	0.9492	0.9498	0.9487	0.9603	0.9605	0.9600
**IoU**	0.9033	0.9036	0.9028	0.9205	0.9206	0.9202

**Fig. 11:**
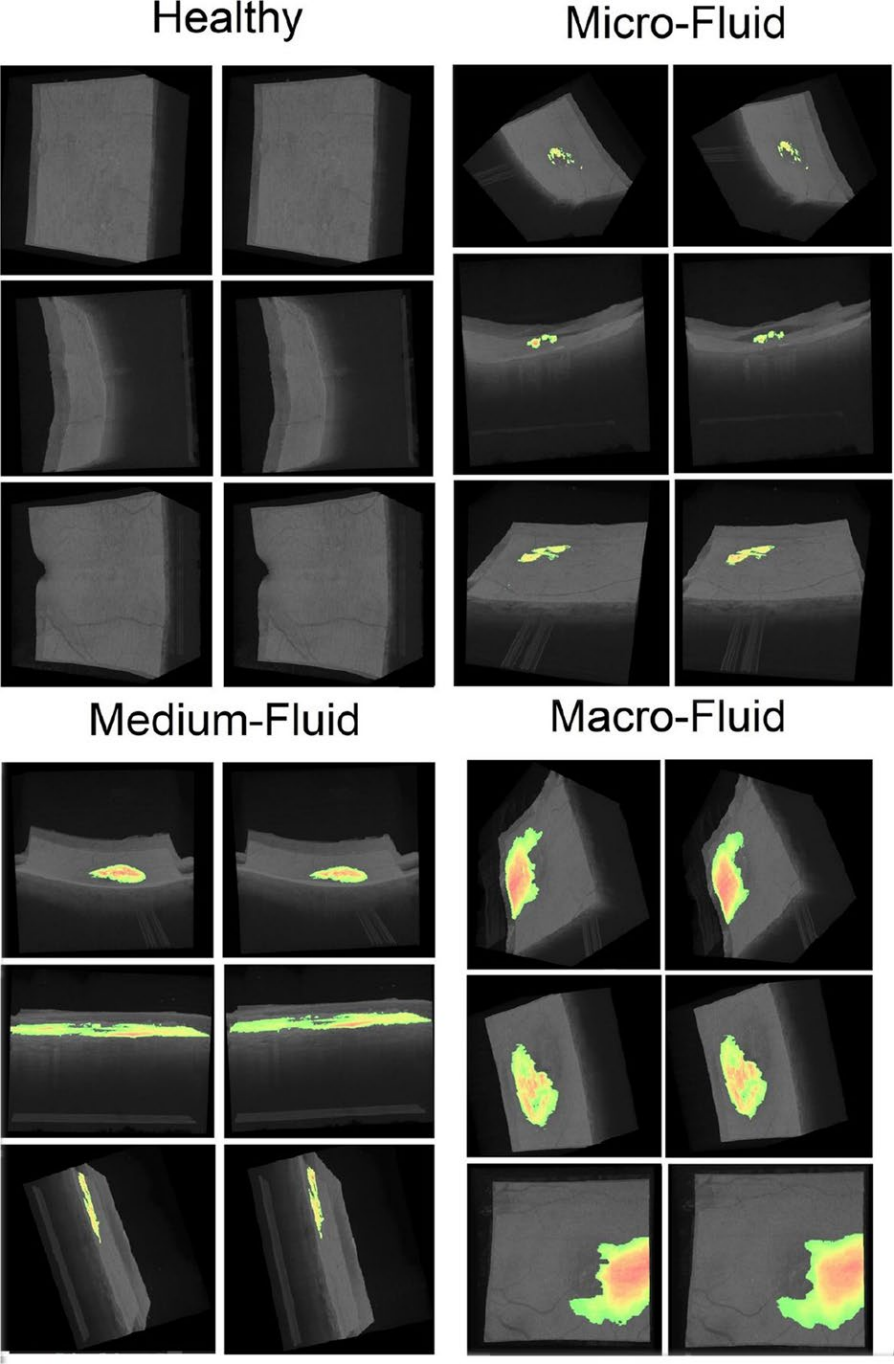
Several representations of the different interactive 3D models generated with the set of volumes of the OCT256 and OCT320 configurations. In each pair of cubes the left image corresponds to the cube created using the masks provided by the expert, while the right image corresponds to the reconstruction using our model

Several representations of the different interactive 3D models generated with the set of volumes OCT256 and OCT320 are presented in Fig. 11. Each 3D model is reconstructed using all the 2D OCT histological sections of each cube and the segmented fluid. Each 3D reconstruction is shown from a different plane and is accompanied by a 3D reconstruction made with the masks created by the experts in order to establish a comparison. The interactive 3D reconstruction allows the physician to rotate, move or zoom the reconstructed 3D volume to observe the distribution of the fluid from any plane that the physician needs. We divided the figure into 4 groups based on the amount of fluid in each cube, thus showing the different levels of extension that can be found. The first one, called healthy, corresponds to the cubes reconstructed with images of healthy patients. The second, third and fourth correspond, respectively, to cubes reconstructed with images of wet AMD patients with little, quite or much fluid.

The 3D reconstruction allows us to assess at a glance whether the patient has the disease or not. It is clearly observed how there is no presence of fluid in the cubes corresponding to healthy patients, while it is observed in the cubes corresponding to patients with wet AMD. Therefore, our proposal offers an useful visualization that helps to diagnose if the patient is developing wet AMD. In addition, as can be seen in the various pathological examples, the 3D visualization allows to quickly quantify how widespread the fluid is, helping the clinician to quickly find out the severity of the pathology and producing a more appropriate treatment decision.

## Conclusions

Nowadays, the development of computational tools that reduce the workload and subjectivity of medical diagnosis is of vital importance. Thus, diseases such as wet AMD need urgent treatment and therefore their early detection is of special interest when developing this type of systems.

In this work, we have presented a methodology to automatically create a 3D visualization of AMD-associated fluid using OCT images. We took advantage of a pre-trained model with a large number of MRI images for a tumor segmentation task to obtain an accurate and robust segmentation model with a reduced number of samples. Thanks to this, we are able to generate a 3D reconstruction and visualization, a powerful tool that helps to diagnose and monitor this disease. Our proposal facilitates and lightens the medical workload by allowing for a quick and repeatable analysis, significantly reducing the impact of subjectivity in the final diagnosis. This promotes an early detection of the disease as well as proper monitoring, a critical factor in preventing the severe, irreversible vision loss that characterizes wet AMD.

As future work, there are several prospects for improving the method and increasing its usefulness for the clinical sector. Although the closing improves the quality of the final visualization, there are other methods capable of producing a similar effect that may work better. Moreover, it would be interesting to explore the possibility of using a three-dimensional segmentation network that uses information from multiple adjacent slices in order to improve the segmentation of our method. This perspective is of particular interest, since it may help with small regions of fluid present in the transition slices to areas where no fluid is present. Finally, it would be interesting to expand the use of this methodology with other ocular diseases in order to create a multifunctional automatic visualization.

## Data Availability

Data and code underlying the results presented in this paper are not publicly available at this time.
